# Recombinant erythropoietin for the anaemia of patients with advanced Gastrointestinal Stromal Tumours (GIST) receiving imatinib: an active agent only in non progressive patients

**DOI:** 10.1186/2045-3329-2-11

**Published:** 2012-09-05

**Authors:** Florence Duffaud, Caroline Even, Isabelle Ray-Coquard, Emmanuelle Bompas, Thanh Khoa-Huynh, Sebastien Salas, Philippe Cassier, Armelle Dufresne, Sylvie Bonvalot, Francoise Ducimetiere, Axel Le Cesne, Jean-Yves Blay

**Affiliations:** 1 Hôpital La Timone, Marseille, France; 2 Institut Gustave Roussy, Villejuif, France; 3 Centre René Gauducheau, Nantes, France; 4 Centre Léon Bérard, Department of Medecine, French Sarcoma Group (GSF-GETO), European Organisation for Research and Treatment of Cancer (EORTC), University Claude Bernard Lyon I, Lyon, France

**Keywords:** GIST, Imatinib, Anaemia

## Abstract

**Abstract:**

Recombinant erythropoietin for the anaemia of patients with advanced Gastrointestinal Stromal Tumours (GIST) receiving imatinib : an active agent only in non progressive patients.

**Background:**

Imatinib is a standard treatment for advanced/metastatic GIST and in adjuvant setting. Anaemia is frequently observed in patients with advanced GIST, and is one of the most frequent side effects of imatinib with grade 3–4 anaemia in 10% of patients. Whether EPO treatment is useful in the management of GIST patients receiving imatinib treatment is unknown.

**Methods:**

A retrospective study of EPO treatment in GIST patients receiving imatinib was undertaken in 4 centres. Thirty four patients received EPO treatment among the 319 GIST patients treated with imatinib in clinical trials or with compassionate use between 2001 and 2003. The efficacy of EPO on the anaemia of patients with GIST treated with imatinib was analyzed.

**Results:**

There were 18 males and 16 females with a median age of 59 years. Median WHO-PS was 1. Primary tumour sites were mainly gastric (32%) and small bowel (29%). Sites of metastases were mainly liver (82%) and peritoneum (79%). The median delay between the initiation of imatinib treatment and EPO was 58 days (range 0–553). Median haemoglobin (Hb) level prior to EPO was 9 g/dL (range 6,9-11,8) and 11,7 g/dL (range 6,8-14,4) after 2 months. An increase of more than 2 g/dL was observed in 18 (53%) of patients. None of the 7 patients who progressed (PD) under imatinib treatment (400 mg/day) experienced HB response, as compared to 66% (18/27) of the remaining patients (PR + SD) (p = 0,002). Primary tumour site, liver metastases, peritoneal metastases, age, gender did not correlate with HB response to EPO. Response to EPO was observed in 2/11 patients receiving high-dose imatinib (800 mg/day) *vs* 16/23 of others. Using logistic regression, only PD before EPO treatment was retained as a predictive factor for EPO response.

**Conclusion:**

EPO enables to increase Hb in most anaemic GIST patients who do not progress under imatinib, but not in patients with progressive disease.

## Introduction

Imatinib (Glivec® / Gleevec®, Novartis) is an orally administered small molecule tyrosine kinase inhibitor active against BCR-ABL, KIT, and PDGF. It has proven to be remarkably effective in gastrointestinal stromal tumours (GIST) patients with advanced/metastatic disease [1-5], as well as in adjuvant phase [6]. Imatinib is approved worldwide for use in GIST, with a usual recommended dose of 400 mg daily [7]. Overall, imatinib is well tolerated, but severe side effects are reported in 2-5% of patients [8]. Anaemia is one of the most frequent side effects observed in imatinib treated patients with GIST (90% of patients), mostly mild to moderate, while this drug is associated with severe grade 3–4 anaemia in less than 10% of patients [8]. Erythropoietin (EPO) is well demonstrated to improve anaemia associated with chemotherapy in solid tumours and quality of life in anaemic patients with cancer [9]. It reduces the need of red blood cell transfusion in cancer patients. Whether EPO treatment is useful in the management of GIST patients receiving imatinib is unknown.

In the present retrospective study, the impact of EPO treatment on the symptomatic anaemia of GIST patients treated with imatinib and the predictive factors of EPO efficacy was investigated.

## Patients and method

### Study group

This retrospective study of EPO treatment in GIST patients receiving imatinib was performed in 4 French centers (Institut Gustave Roussy, Villejuif, Hôpital E. Hérriot, Lyon, Centre Léon Bérard, Lyon, Hôpital La Timone, Marseille). Patients with histologically proven advanced unresectable and/or metastatic GIST characterized by KIT expression (assessed by DAKO assay based on immunohistochemistry), receiving imatinib (Glivec®) in clinical trials or with compassionate use,and who developed anaemia (haemoglobin ≤ 11 g/dL) were treated with EPO. All patients had measurable disease as defined by Response Evaluation Criteria in Solid Tumours (RECIST).

### Treatment plan and dose modifications

Patients were treated for anaemia with weekly doses of 30,000U EPO subcutaneously (epoetin alpha, n = 30, 88% or epoietin beta, n = 2, 6%) or 150 μg/week of darbepoetin alpha (n = 2, 6%) until the haemoglobin (Hb) level increase up to 11–13 g/dL according to the recommendations for clinical use. EPO was discontinued if the Hb levels remained stable at 11–13 g/dL or higher. For patients with suspected iron-deficient anaemia (based on clinical history and red blood cell parameters), levels of serum iron, iron-binding capacity, and serum ferritin were evaluated, as was any possible cause of blood loss, before EPO was considered. Only patients with iron-deficient anaemia received iron supplementation. Serum EPO levels were not screened before the initiation of EPO therapy.

For the purpose of the current analysis, a favourable response to EPO was considered as a sustained (i.e. lasting > 4 weeks) increase in the Hb level ≥ 2 g/dL from the pre-treatment values. Dose reductions of imatinib for non haematological or haematological toxic effects were initiated according to the clinical trials recommendations [3-5]. No dose interruptions or dose reductions were indicated for anaemia alone. Patients were followed with complete blood counts (CBC) weekly during the first 4 weeks of imatinib therapy and then every 2–4 weeks.

### Statistical considerations

A univariate analysis was performed on the following variables: age at diagnosis, sex, site of metastases, initial dose of imatinib, and response to imatinib. Statistical significance was determined using the Fisher exact test. To identify the potential predictive factors of EPO efficacy, a logistic regression model was performed.

## Results

Thirty four patients who developed anaemia (Hb level ≤ 11 g/dL) among the 319 (11%) GIST patients treated with imatinib in clinical trials or with compassionate use in the 4 French centres, between July 2001 and August 2003, received EPO therapy. The median time from the initiation of imatinib to the initiation of EPO was 58 days (range, 0–553 days). 2 patients started imatinib and Epo on the same day. Demographic and clinical characteristics are presented in Table 1. There were 18 males and 16 females with a median age of 59 years (range, 29–85 years) and a median WHO-PS of 1 (range, 0–3). Thirty two patients had a metastatic GIST and two patients had a locally advanced disease. The primary tumour sites were gastric (n = 11, 32%) and small bowel (n = 10, 29%). Of note, a relative large proportion of patients had primary mesenteric or peritoneal GIST (10, 30%). The sites of metastases were mainly liver (82%) and peritoneum (79%). Ten out of 34 (29%) patients required red blood transfusion before starting EPO treatment.

**Table 1:** Clinical characteristics

**Characteristics**	**Patients**
**N (%)**	
**Gender**	
Male	18 (53%)
Female	16 (47%)
**Age** (median, range)	60 (29–85)
**ECOG PS**	
0	2 (6%)
1	17 (50%)
2	12 (35%)
3	3 (9%)
**Tumour site**	
Stomach	11 (32.4%)
Small Intestine	10 (29.4%)
Mesentery	7 (20.6%)
Other	6 (17.6%)
(peritoneum3, colon 1, rectum 1, oesophagus 1)	
**Metastatic sites**	
Liver	28 (82%)
Peritoneum	25 (73.5%)
Lung	4 (12%)
Other	10 (29.5%)
**Transfusion before EPO treatment**	
Yes	10 (29%)/
No	24 (71%)
**Starting Dose of Imatinib/day**	
400 mg	25(67.6%)
800 mg	9(11.7%)

Response to the EPO treatment is presented in Table 2. Median Hb level prior to EPO was 9 g/dL (range, 6.9-11.8 g/dL) and 11.7 g/dL (range, 6.8-14.4 g/dL) after 2 months. Of the 34 patients receiving EPO, 18 (53%) had an increase in Hb levels of ≥ 2 g/dL. Eight additional patients had an increase in Hb levels of 1–1.9 g/dL. Therefore, only 8 of 34 (23.5%) patients had no response at all to EPO therapy. None of the 7 patients who had previously progressed (PD) under imatinib (400 mg/day) obtained an Hb increase after EPO treatment, as compared to 66% (18/27) of the remaining patients (patients with a partial response to imatinib or with a disease stabilization, \PR + SD]) (p = 0,002). There were no differences in responses between the three recombinant EPO used, but there were only 2 patients treated with Epo beta, and darbepoietin respectively. No patient discontinued imatinib therapy because of anaemia. Six non-responding patients to EPO required red blood cells transfusion.

**Table 2:** Haemoglobin response and clinical parameters

**Clinical paramaters**	**Response to EPO, N(%)**		**Chi square**
	**Non Response**	**Response**	**P value**
**Response to Glivec**			
**SD + PR**	9(56.3%)	18(100%)	**0.02**
**PD**	7(44%)	0(0%)	
**Male**	9(56.3%)	9(50%)	0.7
**Female**	7(44%)	9(50%)	
**Liver metastases**			
**No**	1(6.3%)	5(28%)	0.2
**Yes**	15(94%)	13(72%)	
**Lung metastases**			
**No**	14(875%)	16(89%)	1.0
**Yes**	2(12.5%)	2(11%)	
**Peritoneal metastases**			
**No**	3(19%)	6(33.5%)	0.44
**Yes**	13(81.5%)	12(66.5%)	
**Imatinib dose**			
**400 mg/d**	9(56.3%)	16(89%)	**0.05**
**800 mg/d**	7(44%)	2(11%)	
**Age**			
**≥ 60 years**	8(50%)	9(50%)	1.0
**< 60 years**	8(50%)	9(50%)	

° Ficher exact test.

An important question is whether EPO administration is active on the anaemia induced by imatinib, on the anaemia induced by the tumor through iron loss or inflammation, or both. The efficacy of EPO in patients who initiated EPO treatment within the first 2 months of imatinib initiation, between 2 and 6 months and beyond was compared: no significant difference was observed between the three groups (p = 0,3, not shown), indicating that EPO is active also in patients who develop anaemia as a consequence of imatinib treatment,

Primary tumour site, liver and peritoneal metastases, age and gender did not correlate with Hb response to EPO. Response to EPO was observed in 2/9 (22%) patients receiving high-dose imatinib as initial treatment (800 mg/day) versus 16/25 (64%) of others (p = 0.05). Using logistic regression analysis, disease progression (PD) before EPO treatment was the only parameter retained as a predictive factor for EPO response.

## Discussion

Anaemia is a frequently reported haematological side effect in clinical trials with imatinib in GIST [1-4]. To our knowledge, the capacity of EPO to improve the anaemia observed in patients treated with imatinib in GISTs has not been reported, and no article has been reported on this topic within Pubmed (date of access 25/3/2012).

In this study, we investigated the efficacy of EPO treatment on the anaemia of GIST patients treated with imatinib and the predictive factors of EPO efficacy. Hb levels increased in 76.5% of patients following EPO administration in this retrospective study. Of note, somepatients were given EPO with baseline Hb levels above 11 g/dL. This reflects practices which are no longer recommended following the recent clinical practice guidelines regarding EPO administration in cancer patients [10]. EPO was reported as safe by the investigators of this retrospective study, and no severe complications possibly caused by imatinib were reported.

Surprisingly, an over representation of mesenteric GIST is observed in this relatively small series of patients, possibly because of a more aggressive behaviour of primary tumors occurring in these sites, resulting in a greater impact on the general status of the patient.

It has been reported that EPO improves anaemia induced by imatinib therapy in patients with chronic myeloid leukaemia in chronic phase [11]. The present data confirm that EPO is also an efficient treatment in patients with GIST. However, only patients in whom imatinib induces tumor control were found to respond to EPO, and none of the patients progressing under imatinib achieved Hb response to EPO. Importantly, baseline Epo levels were not measured in this series. The role of the paraneoplastic inflammatory syndrome in the resistance to EPO in progressive patients was not investigated either. The impact of iron supplementation in non sideropenic patients, while controversial, was also not explored in this retrospective series, but may also be worth exploring in refractory patients.

For symptomatic and persistent anaemia associated with imatinib, EPO may therefore be considered. Further studies investigating the impact on other measures, such as quality of life and fatigue are needed to measure the benefits of this treatment, even though. Furthermore, the 62005 intergroup phase III trial from EORTC-STBSG [12] in advanced/metastatic GIST patients reported that Hb level is significantly correlated to imatinib response and to progression free survival (PFS) with high imatinib response and favourable PFS predicted by high Hb level. Whether EPO treatment improves imatinib antitumour activity in this context remains to be explored. It must be stressed however that EPO treatment has been reported to impact negatively on survival in a meta-analysis of all Epo trials [10]. The present retrospective study is of course not adequate to address this important question.

It can be concluded that Epo treatment increases Hb level in patients with GIST receiving imatinib with anaemia, whether related to imatinib treatment or to the disease, but that this benefits only to patients achieving tumour control under imatinib treatment.

## Competing interest

JYB, FD, ALC, IRC have received research grants and honoraria from Roche, Novartis, Jansen and Amgen Supported in part by grants from LYRIC, LabEX DEvweCan, and Eurosarc FP-7 278742.

## Authors’ contributions

FlD established the protocol, contributed to obtain academic funding, participated to data collection and analysis, wrote the initial manuscript, and approved the final version. CE participated to data collection and analysis, and approved the final version; IRC participated to data collection and analysis, and approved the final version; EB participated to data collection and analysis, and approved the final version; TKH participated to data collection and analysis, and approved the final version; SS participated to data collection and analysis, and approved the final version; PC participated to data collection, and approved the final version; AD participated to data collection, and approved the final version; SB participated to data collection, and approved the final version; FrDuc participated to data collection, funding, animation of the research group and approved the final version; ALC participated to data collection and analysis, and approved the final version; JYB established the protocol, contributed to obtain academic funding, participated to data collection and analysis, contributed to the different version, and approved the final version. All authors read and approved the final manuscript.
